# Targeting H3K36 methyltransferases NSDs: a promising strategy for tumor targeted therapy

**DOI:** 10.1038/s41392-021-00616-1

**Published:** 2021-06-03

**Authors:** Xuerun Peng, Qian Peng, Lei Zhong

**Affiliations:** 1 Personalized Drug Therapy Key Laboratory of Sichuan Province, Department of Pharmacy, Sichuan Provincial People’s Hospital, School of Medicine, University of Electronic Science and Technology of China, Chengdu, Sichuan China; 2 Radiation Oncology Key Laboratory of Sichuan Province, Sichuan Cancer Hospital & Institute, Sichuan Cancer Center, School of Medicine, University of Electronic Science and Technology of China, Chengdu, China

**Keywords:** Epigenetics, Drug development

Recently, two studies published in Nature identified genetic changes in the nuclear receptor-binding SET domain protein (NSD) family of histone methyltransferases as oncogenic drivers in some malignancies, and revealed the nucleosome-based recognition and histone-modification mechanisms of NSD2 and NSD3.^1,2^

Tumorigenesis and progression are under the control of complex processes governed not only by gene mutation but also by epigenetic dysregulation. As an important branch of targeted cancer therapy, some progress has been made for epigenetic therapies. Thus far, total ten small molecule epigenetic inhibitors are available in the clinic, including two DNA methyltransferase inhibitors (azacytidine and decitabine), five histone deacetylase inhibitors (vorinostat, romidepsin, belinostat, tucidinostat, and panobinostat), two isocitrate dehydrogenase inhibitors (enasidenib and lvosidenib), and one enhancer of zeste homolog 2 inhibitor (tazemetostat). They can significantly prolong the survival of various cancer sufferers, particularly the patients with hematologic malignancies such as chronic myelomonocytic leukemia, acute myeloid leukemia, multiple myeloma, peripheral or cutaneous T-cell lymphoma. Despite this achievement, these approved epigenetic inhibitors just act on a small number of epigenetic regulatory proteins. The mechanisms of action of many epigenetic targets related to tumorigenesis have not been elucidated, which restricts the development of novel anticancer epigenetic therapies. Therefore, a deeper understanding of the mechanisms of epigenetic alterations in malignancies may contribute to further development and optimization of epigenetic therapies for distinct types of cancers.

Amplification of the 8p11-12 genomic region occurs frequently in the tumorigenesis of lung squamous cell carcinoma (LUSC). The study by Yuan et al. identified H3K36 methyltransferase NSD3, rather than FGFR1, as an oncogenic driver within the 8p11-12 amplicon in LUSC.^1^ The authors established a mouse LUSC model harboring canonical LUSC alterations co-occurring with 8p11-12 amplification (constitutively active PI3K, overexpression of SOX2, and deletion of CDKN2A and CDKN2B), named PSC mouse LUSC model. Increased NSD3 expression in lungs tracks with the progression of LUSC in this model. Depleting *NSD3* could significantly restrain tumor growth and extend the lifespan of PSC mice.

**Fig. 1:**
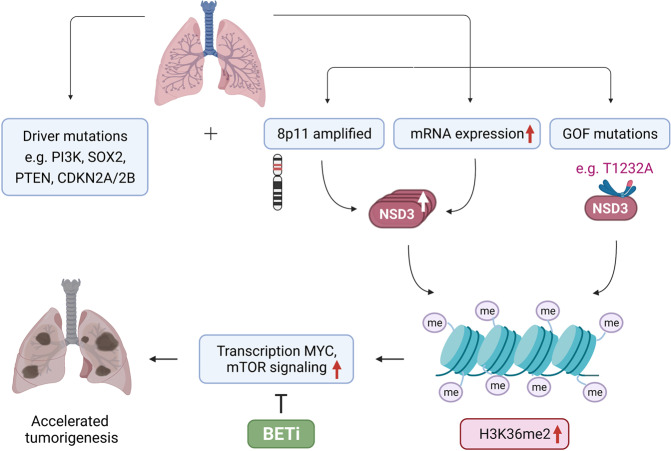
Model of the role for enhanced NSD3 activity in tumorigenesis of LUSC. LUSC tumorigenesis is driven by numerous genetic alternations such as 8p11-12 amplification, constitutively active PI3K, overexpression of SOX2, and deletion of CDKN2A/2B and PTEN. Yuan et al. identified NSD3 amplification as the causative mutation of the 8p11-12 amplicon in LUSC. Amplificaton of NSD3 resulted in elevated NSD3 expression and hence increased dimethylation of H3K36, which cooperates with other driver mutations to boost LUSC pathogenesis. The GOF mutant NSD3 (T1232A) can act like amplified or overexpressed *NSD3* in elevating H3K36me2 expression. Overexpression or GOF mutation of NSD3 increases H3K36me2 synthesis, which stimulates transcription of crucial oncogenic targets including genes involved in MYC-associated pathways and mTOR signaling

Through analyzing the genetic alterations and mRNA expression of *NSD3* in LUSC datasets from The Cancer Genome Atlas (TCGA) and human LUSC cell lines as well as patient-derived xenograft (PDX) samples, Yuan et al. found that NSD3 overexpression could be observed in ~60% of LUSC samples, 20% of which contain 8p11-12 amplification.^1^ In addition, the gain-of-function (GOF) variant NSD3 (T1232A) was also detected in LUSC samples, though it is not as common as *NSD3* amplification (Fig. 1). Among all TCGA-documented variants within the NSD3 catalytic domain, NSD3 (T1232A) has the highest activity on H3K36 dimethylation. Structural dynamic analysis revealed that T1232A mutation changed the local dynamics throughout the functional regions of the NSD3 catalytic domain, relieving its auto-inhibition state and increasing accessibility of H3 substrate. The authors used NSD3 (T1232A) to model the enhanced catalytic activity resulting from amplified or overexpressed *NSD3* in LUSC tumorigenesis. Expression of NSD3 (T1232A) in mouse LUSC models pathologically increased H3K36me2 and the transcription of oncogenes, resulting in accelerated oncogenesis and reduced survival.

Furthermore, the tumorigenesis-promoting effect of NSD3 was also verified in human LUSC cells, particularly in LUSC PDX samples harboring *NSD3* amplification or T1232A mutation. NSD3 depletion elicited prominent inhibition of tumor growth in these PDX models. Notably, the authors discovered that NSD3-driven LUSC tumorigenesis was more sensitive to bromodomain and extra-terminal (BET) inhibitors (Fig. 1).^1^ This is consistent with the previous studies, in which depleting NSD3 could intensively sensitize acute myeloid leukemia cells to BET inhibitors.^3^ These findings offer a potential strategy for the treatment of NSD3-driven malignancies and may also expand the indication of BET inhibitors.

In the same issue of Nature, a simultaneous study by Li et al. solved the cryo-electron microscopy structures of NSD2 and NSD3 as well as their cancer-related hyperactive variants bound to mononucleosomes.^2^ They found that binding of NSD2 or NSD3 to the nucleosomes unwrap the DNA near the linker region, which makes the catalytic core insert between the unwrapped DNA and histone octamer. The network of histone and DNA-specific contacts between NSD2/3 and the nucleosome accurately locates the position of the enzyme on the nucleosome, which explains the specificity of NSD2/3 to H3K36 methylation.

Biochemical analysis in this study showed increased potency of cancer-related variants of NSD2 (E1099K or T1150A) and NSD3 (E1181K or T1232A) in dimethylating H3K36 compared with wild-type proteins.^2^ Further structural analysis elucidated the molecular basis for the enhanced activities of these mutants. For Lys1099 of NSD2, the E1099K mutation changes the negatively charged amino acid (Glu) at position 1099 to a positively charged (Lys) residue, which partners with Lys1124 and hence raises the electrostatic interactions between NSD2 and the nucleosome, resulting in elevated activity.^2^ For Thr1232 of NSD3, the T1232A mutation causes the formation of a pair of novel hydrogen bonds. The newly formed interactions facilitate insertion of H3K36 into the NSD3 catalytic pocket, thus enhancing the catalytic activity. The corresponding T1150A mutation in NSD2 shares a similar mechanism of NSD3 (T1232A). To test the activity of these GOF mutants in cancer cells, Li et al. depleted NSD2 and NSD3 in human bone osteosarcoma cell line U2OS and head and neck squamous cell carcinoma (HNSCC) cell line UD-SCC-2, respectively, which led to decreased H3K36me2 expression and cell proliferation. In contrast, complementation with NSD2 or NSD3 GOF mutations boosted tumor cell growth.^2^

Taken together, these two studies exhibited the growth-promoting effects of NSD2 and NSD3 in several tumors, particularly in the tumorigenesis of LUSC. Given the high frequency of 8p11-12 amplification or GOF mutations of NSDs in many other malignancies such as breast cancer and HNSCC,^4,5^ targeting H3K36 methyltransferases NSDs can not only benefit LUSC treatment. NSDs are promising targets for tumor targeted therapy. Furthermore, Yuan et al. found the increased sensitivity of NSD3-regulated LUSC to BET inhibitors and Li et al. solved the structure of NCP-bound NSD proteins and clarified the molecular mechanisms. These findings provide strong supports for the further development of drugs targeting NSDs and the optimization of therapeutic strategies for treating NSD-driven malignancies.
